# **“**Raising the curtain on the equality theatre”: a study of recruitment to first healthcare job post-qualification in the UK National Health Service

**DOI:** 10.1186/s12960-022-00754-9

**Published:** 2022-07-08

**Authors:** John Hammond, Nigel Davies, Elizabeth Morrow, Fiona Ross, Tushna Vandrevala, Ruth Harris

**Affiliations:** 1 Centre for Allied Health, St George’s University of London, Cranmer Terrace, Tooting, London, SW17 0RE UK; 2 College of Health, Medicine and Life Sciences, Brunel University London, Kingston Lane, Uxbridge, UB8 3PH UK; 3 Research Support NI, Downpatrick, Northern Ireland; 4 Emerita Professor, Health and Social Care, Kingston University and St George’s University of London, Cranmer Terrace, Tooting, London, SW17 0RE UK; 5 Centre for Health and Social Care Research, Kingston University and St George’s University of London, Cranmer Terrace, Tooting, London, SW17 0RE UK; 6 Florence Nightingale Faculty of Nursing, Midwifery and Palliative Care, King’s College London, James Clerk Maxwell Building, 57 Waterloo Road, London, SE1 8WA UK

**Keywords:** Healthcare workforce, Recruitment, Human resource management, Workforce diversity, Social justice, Race equality, Equity, Job success, Careers, Employability

## Abstract

**Background:**

UK equality law and National Health Service (NHS) policy requires racial equality in job appointments and career opportunities. However, recent national workforce race equality standard (WRES) data show that nearly all NHS organisations in the UK are failing to appoint ethnically diverse candidates with equivalent training and qualifications as their white counterparts. This is problematic because workforce diversity is associated with improved patient outcomes and other benefits for staff and organisations.

**Aim:**

To better understand the reasons behind underrepresentation of ethnically diverse candidates in first NHS healthcare jobs post-qualification and to identify any structural or systemic barriers to employment for such groups.

**Methods:**

The study was informed by critical theory and the authors’ interdisciplinary perspectives as educators and researchers in the healthcare professions. Data collected from semi-structured face-to-face interviews with 12 nurse and physiotherapy recruiting managers from two NHS trusts in London were analysed using a healthcare workforce equity and diversity conceptual lens we developed from the literature. Using this lens, we devised questions to examine six dimensions of equity and diversity in the interview data from recruiting managers.

**Results:**

Recruiting managers said they valued the benefits of an ethnically diverse workforce for patients and their unit/organisation. However, their adherence to organisational policies for recruitment and selection, which emphasise objectivity and standardisation, acted as constraints to recognising ethnicity as an important issue in recruitment and workforce diversity. Some recruiting managers sense that there are barriers for ethnically diverse candidates but lacked information about workforce diversity, systems for monitoring recruitment, or ways to engage with staff or candidates to talk about these issues. Without this information there was no apparent problem or reason to try alternative approaches.

**Conclusion:**

These accounts from 12 recruiting managers give a ‘backstage’ view into the reasons behind ethnic inequalities in recruitment to first healthcare job in the UK NHS. Adherence to recruitment and selection policies, which aim to support equality through standardisation and anonymisation, appear to be limiting workforce diversity and creating barriers for ethnically diverse candidates to attain the jobs that they are trained and qualified for. The Healthcare Workforce Equity + Diversity Lens we have developed can help to ‘raise the curtain on the equality theatre’ and inform more inclusive approaches to recruitment such as contextualised recruitment or effective allyship between employers and universities.

**Supplementary Information:**

The online version contains supplementary material available at 10.1186/s12960-022-00754-9.

## Introduction

In the United Kingdom (UK) providing fair and equitable opportunities for employment in the National Health System (NHS) is enshrined in Equality Law and NHS mandates for racial equality [1]. Evidence shows that an ethnically diverse healthcare workforce benefits staff, patients, and health systems through improved quality of care [2, 3], access, choice, and satisfaction [4], as well as providing a wider talent pool and more sustainable workforce supply [3], increased efficiency in services, productivity, and innovation [5], cost saving, reduced staff sickness and formal disciplinary processes [6]. Recruitment into the first destination job in the NHS after completing training can be likened to an audition for a role in the theatre. Therefore, exploring recruitment can shine a light on the formal processes and informal aspects, including “how bias might be mitigated, accountability ensured, and a national repository of good practice created” [6].

Readers may be aware of Erving Goffman’s use of the theatre metaphor in dramaturgy theory which helps to explain social aspects of human behaviour [7] (Goffman 1990). The notions of ‘front stage’, ‘backstage’ and ‘off stage’ describe symbolic interactions of self-identification and role adoption, according to whether people are conscious or unconscious of their behaviours or being observed by others. For example, the various behaviours a person might adopt in their public, professional or private lives. Our development and use of the notion of the ‘equality theatre’ is somewhat different. Here, we are focusing on the theatre itself (structures, processes, systems—which Goffman refers to as the ‘setting’) rather than focusing on the roles or behaviours that an individual might chose to adopt. We are interested in the inequalities and barriers that the theatre may create. Indeed, how some individuals may not even make it onto the ‘stage’ to be able to perform. Goffman’s later work did identify two types of character traits in people: those who adapt themselves and those that adapt their surroundings. He theorised that successful people know how to adapt themselves and they know how to adapt the circumstances to achieve their objectives. This is an interesting perspective which resonates with two issues we are exploring here: the idea of candidates ‘fitting in’ to NHS organisations and whether the actors we are focusing on (recruiting managers) have the ‘know how’ to challenge or change the equality theatre.

The positive impact of healthcare workforce diversity on health inequalities and social determinants of health is recognised internationally [8]. The benefits are made possible through various mechanisms that include improvements in organisational learning [9], more culturally competent care [10], and better multicultural health [3]. Developing workforce diversity through recruitment to the NHS workforce is therefore viewed as a clinically and socially significant priority issue for staff teams and patient care.

So, what is the scale of the issue in the UK? The UK NHS Workforce Race Equality Standard (WRES) requires NHS trusts to measure their performance annually against indicators of equality, which include job appointment success [11]. WRES data show that in March 2021, 22.4% (309,532) of staff working in NHS trusts in England were from ethnically diverse backgrounds—an increase from 18.1% in 2017, however White applicants were 1.61 times more likely to be appointed from shortlisting than ethnically diverse applicants. This is the same as 2020 and worse than in 2019 (1.46) and there has been no overall improvement over the past six years. For those who do gain employment, ethnically diverse staff are more likely to be working in jobs at lower pay grades [12] with higher levels of associated health risks [13], discrimination and gendered racism [14].

Racial discrimination is known to begin with the pipeline to professional training [15], continue in applications and selection to the NHS [11], through to inequalities in experiences and opportunities when employed [12]. Understanding of what actually works to support workforce diversity, at all career levels, is currently limited to certain fields or clinical specialisms, for example, emergency medicine [16] and oncology [17]. Investment in successfully widening access and participation to further and higher education is failing to translate into equitable job opportunities for graduating students [18].

In looking at these issues it is important to be clear about the definitions and key concepts associated with workforce race equality. The terms we are using are explained in Box 1. These are definitions that we have adopted for the purpose of this study and article and are grounded in the literature. However, we recognise that alternative terms and understandings may be more suitable and customary in international contexts where alternative models of health care provision and recruitment exist.

Box 1: Key concepts in workforce race equality

**Table:** 

*Recruitment* A set of policies and practices designed to support the appointment of candidates to jobs in an organisation. Various approaches include employer-led selection, allocation of jobs, targeting of specific types of candidates (headhunting), or transitional positions such as placements, internships, volunteering, or apprenticeships that lead to paid appointment. Appointments might be prearranged, guaranteed, or awarded by fulfilling training criteria or by competitive selection. Choices between candidates could be based on applications, interviews, technical or group exercises, algorithms, or other criteria such as evidence of competencies or experiences. Contextualised recruitment is an approach that aims to recognise the diverse skills and experiences candidates might bring to a role or organisation [19]
*Workforce diversity* In a healthcare context, workforce diversity means that a health provider’s employment practices shape an inclusive workplace for people of “visible characteristic of difference”, different gender, age, religion, race, ethnicity, cultural background, sexual orientation, languages, and education [12]. Improving workforce diversity relies on creating a more diverse and accepting culture [20], which values migrants and ethnically diverse groups for their own unique assets, skills, attributes, and potential [11]. An ethnically diverse workforce is known to support sustainable staffing, enrich the quality of patient care, improve staff morale, and facilitate organisational change and innovation in practice through a more accepting organisational culture [6, 8, 9]
*Workforce race equality* Action to ensure employees from ethnically diverse backgrounds have equal access to career opportunities and receive fair treatment in the workplace is enshrined in Equality Legislation. In the UK NHS providers are mandated to show progress against a number of indicators of the Workforce Race Equality Standard (WRES) [11] based on evidence that a motivated, included, and valued workforce helps deliver high-quality patient care, increased patient satisfaction and better patient safety [6, 8, 9]. Race equality aims to reduce bias, prejudice, and discrimination to ensure there is parity in the processes and outcomes [12]
*Social justice* Specific individuals and groups may encounter systemic, attitudinal, and physical barriers to equality because of their personal circumstances and visible characteristics [20]. Social justice describes a belief, action, or movement, that aims to address inequalities by re-distribution or creation of resources, policies, opportunities, or privileges, to achieve fairer societies [20]. In a workforce context, social justice is based on the concepts of human rights and equality [11] and can be defined as the way in which human rights are manifested in the everyday working practices of an organisation
*Equity* The difference between equality and equity must be emphasised. Although both promote fairness, equality aims to treat everyone the same regardless of need, while equity treats people differently dependent on their specific needs [5]. This ‘targeted additionality’ may be the key to reaching equality because diverse individuals get what they need rather than generic or universal solutions. Equity provides everyone with an opportunity to reach their full potential and have an equal chance to live their life as they choose. In the context of recruitment, equity focuses on ‘closing the equality gap’ and can include, for example, the use of information, evidence-based practice, or data, strategies to offer differentiated support to specific groups [5, 6], strategies for promoting inclusion, and targeted approaches to engage underrepresented or marginalised groups, or widen access to job opportunities

## Aims

The aims of this study are to better understand the reasons behind underrepresentation of ethnically diverse candidates in first NHS healthcare jobs post-qualification and to identify any structural or systemic barriers to employment for such groups.

To put these aims in context, an employer-led open market approach is the current standard approach in NHS trusts in the UK. Historically it has not always been this way in nursing or other healthcare professions. Current recruitment practices in the healthcare professions differs from recruitment to medical posts in the UK, which use an allocation model for first job after degree to provide a 2-year foundation course. The medical recruitment model is not perfect as it creates challenges for some graduates who are less able to travel or to relocate near to their allocated post. In contrast recruitment to posts in nursing and AHPs (including physiotherapy, occupational therapy, radiography, speech and language therapy) are undertaken by NHS recruiting managers who are senior clinicians dealing with large numbers of applicants while balancing clinical pressures.

**Fig. 1:**
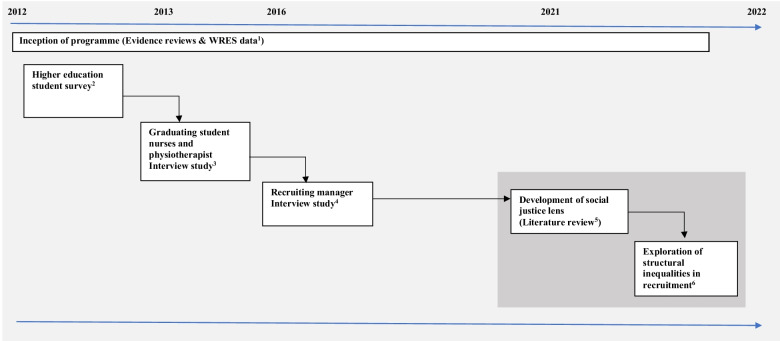
The study in context. Overview of programme of employability research (2012–2022)

The present study was designed and undertaken in the context of a lack of progression, and in some NHS provider organisations a worsening, in race equality in recruitment [12]. There had been a call from WRES for urgent action to prevent further deterioration [6]. As researchers on a programme of (first in kind) employability studies (co-authors JH, ND, RH, FR) we were aware and had published [21–23] findings on statistically significant ethnic inequalities in nurse job seeking success [22]. The elements and methods of the employability programme we will refer to are illustrated by Fig. 1 (Box 2). Our findings on employment inequalities in London trusts have had impact and reach in that they informed the design of WRES measures [6]. We had also used qualitative research to explore student perspectives of ethnic inequalities in physiotherapy and nursing student experiences of job seeking and found students from ethnically diverse backgrounds generally blamed themselves for failing to attain jobs or perceived themselves as not ‘fitting in’ [23].

As educators of university students (JH, ND, TV, RH, FR) in the healthcare professions we also felt a sense of injustice and a moral obligation to ensure all of our students can access the employment opportunities that they have been trained and are qualified for. From our various research and educator roles, we also saw this as an issue of partnership and communication between the university and employers, and a need to smooth the transition from being a student to being an employee. We recognised that students already spend many hours of training in NHS practice placements and have invested financially and emotionally in the idea of becoming a practising healthcare professional. In this sense, the interview process was in a very real way, an audition for that all important first job [21].

Box 2: Overview of methods used in programme of employability research

**Table:** 

**Fig. 2:**
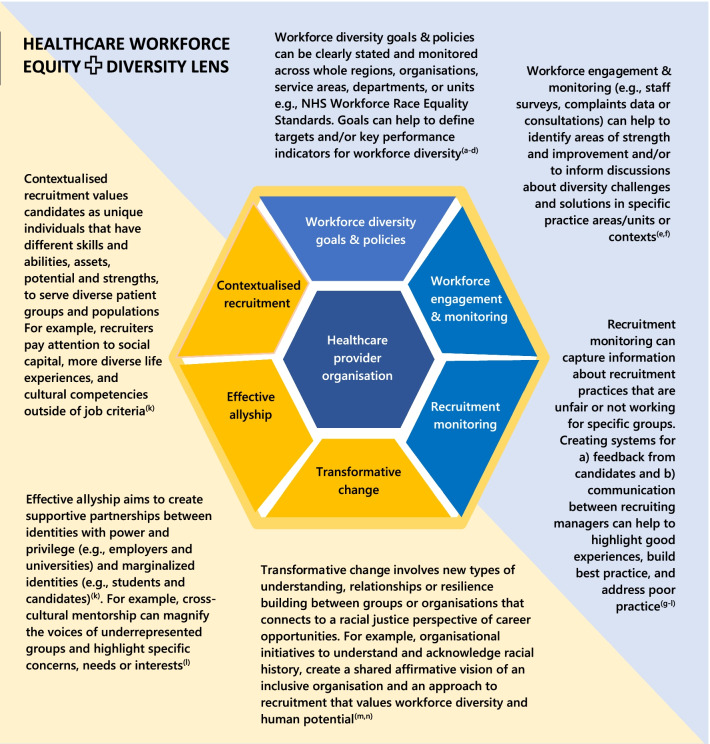
Healthcare workforce equity + diversity lens. Key sources: **a** The nonprofit association of oregon equity & inclusion lens guide. **b** A voice for the reduction of poverty Nashville. **c** philanthropic initiative for racial equity **d** Race Matters Institute. **e** Be Loved Community Equity Lens Map. **f** The Salvation Army Racial Equity Lens. **g** NHS Workforce Research Equality Standard and Equality Delivery System. **h** The Aspen Institute Education & Society Program Racial Equity Lens. **i** Government Alliance on Race and Equity Racial Equity Toolkit. **j** Housing Development Consortium Race Equity Toolkit. **k** Albany Medical College medical allyship module. **l** University of Kent effective allyship resources. **m** The Impact Foundry. **n** Access, Equity, and Acceleration Unit equity lens (see Additional file 2, bibliography for weblinks)

*Evidence reviews and WRES data* Since the inception of this programme of employability research, in 2012, we have systematically searched the academic literature (using MEDLINE, CINAHL, PsycINFO and EMBASE) for studies that examined the equality of employment opportunities for newly qualified healthcare professionals. None have been found online. Our work remains the only research we are aware of, internationally, to highlight ethnic inequality in first job success, racial differences in confidence and preparedness for job seeking, and the behaviour modification strategies job seekers from ethnically diverse backgrounds adopt (“being proactive”, “fitting in”, pre-empting discrimination) to attain a job in unfair systems.
*Higher education student survey* An exploratory study using structured questionnaires and secondary analysis of data routinely collected by the universities about students and their progress during their course. The study was conducted in eight universities within a large, multicultural city in the UK as part of the ‘Readiness for Work’ research programme. Participants were 804 newly qualified nurses who had successfully completed a diploma or degree from one of the universities; a response rate of 77% representing 49% of all graduating students in the study population. Data were collected by self-completed semi-structured questionnaires administered to students at the time of qualification and at three months post-qualification. Routinely collected data from the universities were also collected. Fifty two percent of participants had been offered a job at the point of qualification (85% of those who had applied and been interviewed). Of these, 99% had been offered a nursing post, 88% in the city studied, 67% in the healthcare setting where they had completed a course placement. 44% felt ‘‘confident’’ and 32% ‘‘very confident’’ about their employment prospects. Predictors of employment success included ethnicity, specialty of nursing and university attended. Predictors of confidence and preparedness for job seeking included ethnicity, nursing specialty, gender, and grade of degree. Newly qualified nurses from non-White/British ethnic groups were less likely to get a job and feel confident about and prepared for job seeking.
*Graduating student nurses and physiotherapist interview study* This qualitative study explored the experience of student nurses (n = 12) and physiotherapists (n = 6) throughout their education and during the first 6 months post-qualification to identify key experiences and milestones relating to successful employment particularly focusing on the perspectives from different ethnic groups. Participants were purposively sampled from one university to ensure diversity in ethnic group, age, and gender. Using a phenomenological approach, in-depth semi-structured interviews were conducted at course completion and 6 months later. Two main themes were identified. The ‘proactive self’ (‘It’s up to me’) theme included perceptions of employment success being due to student proactivity and resilience; qualities valued by employers. The second theme described the need to ‘fit in’ with organisational culture. Graduates described accommodating strategies where they modified aspects of their identity (clothing, cultural markers) to fit in. At one extreme, rather than fitting in, participants from minority ethnic backgrounds avoided applying to certain hospitals due to perceptions of discriminatory cultures, ‘I wouldn’t apply there ‘cos you know, it’s not really an ethnic hospital’. In contrast, some participants recognised that other graduates (usually white) did not need to change, and aspects of their identity brought unsolicited rewards ‘if your face fits then the barriers are reduced'.
*Recruiting manager interview study* Confidential one-to-one interviews and a non-judgemental approach were applied in this qualitative study to understand the complexities of race equality issues in NHS contexts. Data were collected in two NHS London trusts in 2015 using semi-structured face-to-face interviews. An interview schedule was designed to incorporate questions about recruitment and selection processes, the role of the recruiting manager, and awareness of ethnic inequalities in job success to explore the sensitive issues surrounding ethnic inequalities in recruitment. A purposive sample of participants was selected according to NHS trust, recruiting manager role to clinical jobs at post-qualification level (i.e. local/unit clinical managers not Human Resource or corporate managers), and consent to participate. Participants were 9 nurse recruiting managers (bands 7-8c) and 3 physiotherapy recruiting managers (bands 8a-c). Interviews lasted between 18–56 min, were audio recorded. Thematic analysis.
*Development of the Healthcare Workforce Equity + Diversity Lens* To develop the lens, we undertook structured searches for evidence in the literature, online information (e.g., institutional reports), and policy papers relating to the concepts of workforce diversity, social justice, and equity. We identified key search terms from our initial readings of the literature and refined these as a group. We undertook multiple searches of WebofScience, PubMed and Google Scholar, (Nov 11, 2021, and Jan 11, 2022) using search terms relating to the concepts ‘workforce diversity’ ‘social justice’ and ‘equity’. Searches included primary research, opinion articles, and reviews published in the English language between 2000 and the search date. Web-based organisational information (policy, corporate, public, and voluntary sector reports, or webpages) was searched using key words (“social justice” “equity” and “workforce diversity”). A pragmatic selection process in this developing area of knowledge and research, was to sift through first 500 returns. Information from 90 items was retrieved, scrutinized against criteria for quality (article source, underpinning evidence) and validity (relevance to aims, consistency with emerging themes [32]). We defined inclusion criteria based on our aims to understand the reasons behind inequalities in employment success and the barriers to first healthcare job. Information from included items (see bibliography, Additional file 2) were extracted to Microsoft Word, synthesized into issues and relevant phrases or concepts, and developed into categories using an inductive thematic approach [32]. We discussed these categories and further refined them to define the six dimensions of the lens (Fig. 2)
*Exploration of structural inequalities in recruitment* The study was informed by critical theory and the authors’ interdisciplinary perspectives as educators and researchers in the healthcare professions. Data collected from semi-structured face-to-face interviews with 12 nurse and physiotherapy recruiting managers from two NHS trusts in London [3] were analysed using the healthcare workforce equity and diversity conceptual lens we developed from the literature [5]. Using this lens, we devised questions to examine six dimensions of equity and diversity in the interview data from recruiting managers

## Methods

### Critically informed approach

In this study, we took a critical stance on the issues. We drew on critical theory [24, 25] and our understandings of how structural inequalities can operate within higher education contexts [15, 26] and the challenges some students face in the transition to healthcare jobs [21–23]. The approach included drawing on critical perspectives from social justice (described in Box 1) and critical race theory (positionality, agency, and power [24, 25]) and locating our claims in theory, best evidence, and UK NHS policies for race equality [11]. Definitions of these key concepts we have drawn on are provided in Box 1. There are substantial literatures bases in each of these areas and we have drawn selectively on theoretical perspectives that include Fraser’s (1995) framings of justice as affirmative and transformative [27], Kaiser et al.’s (2013) observations of the ironic effects of organisational diversity structures [28], Patton’s (2010) developmental evaluation for organisational change [29], and Belle et al.’s (2000) assertions about mentoring for work and careers [30]. Essentially, social justice (described in Box 1) was important for our understanding of the ways that specific individuals and groups, in this context candidates applying for first job, may encounter systemic, attitudinal, and physical barriers to employment.

With this critically informed approach, our research questions focused on understanding (i) the reasons behind racial inequalities in employment success for ethnically diverse groups; (ii) the barriers to employment that ethnically diverse candidates face. These questions focused on the systemic and structural reasons and barriers rather than racism or discrimination at the individual level acknowledging that these forms of discrimination can be interlinked (see Box 1). It is important to note that an assumption we are making in taking this approach is that current recruitment practices are contributing to inequalities in employment opportunities for ethnically diverse candidates. Therefore, the study looks at this assumption and the ‘acts that are performed’ by recruiting managers in relation to recruitment to first job post–qualification, but also ‘the nature of the stage’ on which these acts are performed.

### Development of the Healthcare Workforce Equity + Diversity Lens

Building from this critical stance, we undertook broad yet structured searches for evidence in the literature, online information (e.g., institutional reports), and policy papers relating to the concepts of workforce diversity, social justice, and equity. We identified key search terms from our initial readings of the literature and refined these as a group (Additional file 1). We undertook multiple searches of WebofScience (a platform which enables simultaneous cross-searching of various citation indexes and databases including MEDLINE, CINAHL, PsycINFO and EMBASE) PubMed and Google Scholar, between Nov 11, 2021, and Jan 11, 2022, using search terms relating to the concepts ‘workforce diversity’ ‘social justice’ and ‘equity’ (see Additional file 1 for Medical Subject Headings and key words). We searched for primary research, opinion articles, and reviews published in the English language between 2000 and the search date. Web-based organisational information (policy, corporate, public, and voluntary sector reports, or webpages) using key words (“social justice” “equity” and “workforce diversity”). A pragmatic selection process in this developing area of knowledge and research, was to sift through first 500 returns. Information from 90 items (Additional file 2, bibliography) was retrieved, scrutinised against criteria for quality (article source, underpinning evidence) and validity (relevance to aims, consistency with emerging themes [31]).

We defined inclusion criteria based on our aims to understand the reasons behind inequalities in employment success and the barriers to first healthcare job. Information from included items (see bibliography, Additional file 2) were extracted to Microsoft Word, synthesised into issues and relevant phrases or concepts, and developed into categories using an inductive thematic approach [31]. We discussed these categories and further refined them to define the six dimensions of the lens (Fig. 2).

### Recruiting manager interview participants

In the NHS recruiting managers are responsible for implementing organisational recruitment and selection policy. These responsibilities include analysis of vacant jobs and new posts, job descriptions/person specification, Agenda for Change banding (AfC is the national pay system for all NHS staff), job advertisement, fair assessment of applications and appointments. They are required to undertake training in recruitment and selection processes, ensure interview notes are factual, clear, and legible, make offers of appointment, give candidates feedback, and ensure new staff receive an induction. In order to support the recruitment of a sample of recruiting managers we chose to use confidential one-to-one interviews [32] and use a non-judgemental approach [32] to understand the complexities of race equality issues in NHS contexts. We gained ethics approval and R&D approval in two NHS trusts in London. Data were collected in 2015 using semi-structured face-to-face interviews [33]. An interview schedule was designed to incorporate questions about recruitment and selection processes, the role of the recruiting manager, and awareness of ethnic inequalities in job success to explore the sensitive issues surrounding ethnic inequalities in recruitment.

A purposive sample [31] was selected according to NHS trust, recruiting manager role to clinical jobs at post-qualification level (i.e. local/unit clinical managers not Human Resource or corporate managers), and consent to participate. We aimed to recruit 12 recruiting managers in nursing and physiotherapy (influenced by the Access Agreement funding). The sample was achieved by developing recruitment materials (participant information letter) and consent process to dispel any fear of accusations of racism. Participants were 9 nurse recruiting managers (bands 7-8c) and 3 physiotherapy recruiting managers (bands 8a-c) (Table 1). Interviews lasted between 18 and 56 min, were audio recorded and transcribed verbatim. As the issues are sensitive, we have protected participant confidentiality by not naming individuals or the NHS trusts.

**Table 1:** Recruiting manager participants

Int Ref	Role	Band	Ethnicity	Male/female	Institution	Interview coder
Int 1	Nurse manager	7	Black	F	NHS trust 1	RH/ND
Int 2	Nurse manager	7	White	M	NHS trust 1	RH
Int 3	Physio manager	8B	White	M	NHS trust 1	RH
Int 4	Nurse manager	8A	White	F	NHS trust 1	RH
Int 5	Physio manager	8A	White	F	NHS trust 1	RH
Int 6	Nurse manager	7	Black	F	NHS trust 1	RH
Int 7	Nurse manager	8A	Asian	F	NHS trust 2	ND
Int 8	Nurse manager	8C	White	M	NHS trust 2	ND
Int 9	Nurse manager	8A	Asian	M	NHS trust 2	ND
Int 10	Physio manager	8C	White	F	NHS trust 2	ND
Int 11	Nurse manager	8A	White	F	NHS trust 2	ND
Int 12	Nurse manager	8A	Black	F	NHS trust 2	ND

Codes used in text:

[Int1: NRM band 7 Black F]

[Int2: NRM band 7 White M]

[Int3: PRM band 8 White M]

[Int4: NRM band 8 White F]

[Int5: PRM band 8 White F]

[Int6: NRM band 7 Black F]

[Int7: NRM band 8 Asian F]

[Int8: PRM band 8 White M]

[Int9: NRM band 8 Asian M]

[Int10: NRM band 8 White F]

[Int11: NRM band 8 White F]

[Int12: NRM band 8 Black F]

### Analysis

An initial thematic analysis of the recruiting manager interview data [31] (by ND and RH, with contributions from JH and FR) pursued issues about candidate attributes associated with inequalities in job success [22], ‘getting in’ and ‘fitting in’ [23], favouritism [34], and organisational factors associated with workforce race equality [11]. The thematic approach provided an insight into the perspectives of recruiting managers, however, analysing the data thematically failed to locate the perspectives in a critical framework that might help to explain the reasons for racial inequalities in job success beyond the level of individual practice. That is, until the development of the Healthcare Workforce Equity + Diversity Lens, we lacked a framework to interrogate and interpret barriers to employment beyond individual interactions between recruiting managers and candidates.

In 2021 the team, who were now spread across four London universities, was invited to reconvene (by FR). This reengagement with the data was prompted by new understandings of systemic racial injustice emerging from COVID pandemic and BlackLivesMatter human rights campaigns. With a small research development grant from the host university (GBP4K), two new members (TV and EM) were invited to work on a critically informed analysis of the interview data. These members brought new perspectives from their work on social justice, inclusion (EM) and inequalities research (TV). The team met online and familiarised themselves with the issues and intentions for the study. RH explained the evidence on ethnic inequalities in first job success that had been found in the student surveys and how this knowledge had informed the interview questions, listed in Fig. 1. We individually read the interview data (12 anonymised transcripts) and the previous unpublished thematic analysis. Following the meeting EM created a list of the equity and diversity issues/themes we had discussed. Sharing these themes with ND and JH resulted in us further developing these ideas into a conceptual lens for the study which drew on equity and diversity perspectives.

In terms of the critical re-analysis, Developing the Healthcare Workforce Equity + Diversity Lens was key to understanding the reasons behind the systemic inequalities in first job success that we had suspected as senior leaders (former Dean) and academic lecturers informed by our relationships with trust managers, graduating students and colleagues working in the NHS, and our previous student surveys had proven (detail in Fig. 1). The next step in the analysis was to use the lens to analyse the recruiting manager interview data. We did this by devising questions that directly reflect the dimensions of the lens, as follows:

1. Do recruiting managers express clear goals and policies, targets, or performance indicators for workforce diversity? (Are these personal, unit, organisational, or other?)
2. Are recruiting managers involved in, or aware of, any workforce engagement and monitoring of diversity, or actions to identify or address diversity issues?
3. Are recruiting managers aware of, or engaged in, any monitoring or assessment of ethnic diversity in recruitment?
4. How far do recruiting managers value candidates as unique individuals that have different skills and abilities, assets, potential and strengths, to serve diverse patient groups and populations?
5. How are the interests or concerns of underrepresented ethnically diverse groups heard, voiced, or advocated for in relation to recruitment practices?
6. How far do recruiting managers see a need for change, better understanding, relationships, or resilience building between groups or organisations to support fairer recruitment and retention of human potential?

We ensured that the assumptions and claims made in the analysis were re-examined through this reflexive iterative process [31] which included questioning the data and questioning the interpretation we were making. This approach, using questions derived from the lens, helped to establish rigour and confirmability of the results and the links back to the interview data [31]. As theoretically informed and evidence-based analytic tools, the lens and six questions, helped to keep our focus on revealing the structural and systemic barriers to employment success [32]. They also helped us to avoid recasting unhelpful accusations of recruiter racism or candidate inadequacy at the individual level [34], which has so far hindered meaningful organisational progress on race equality [12].

## Results

The results are presented according to the six critically informed questions above. Issues identified in the data are summarised by Table 2 and illustrated with verbatim quotes from recruiting manager participants in nursing (abbreviated to NRM) and physiotherapy (abbreviated to PRM). The ethnicity (White, Black, Asian) and gender (male/female) of each participant is indicated after each quote, and in Table 2. We are using these broad categories to protect participant confidentiality; however, we acknowledge the limitations of these classifications for conveying how participants may perceive and choose to express their own cultural heritage, ethnicity or personal or professional identity [24, 25], or how such identities may differ depending on the various public and private ‘stages’ these actors are operating on.

1. Do recruiting managers express clear goals and policies, targets, or performance indicators for workforce diversity? (Are these personal, unit, organisational, or other?)

**Table 2:** Critically informed analysis

Questions derived from the Healthcare Workforce Equity + Diversity Lens	Emerging issues for healthcare workforce recruitment	Illustrative quotes from recruiting managers in nursing (NRM) and physiotherapy (PRM)
1) Do recruiting managers express clear goals and policies, targets, or performance indicators for workforce diversity? (Are these personal, unit, organisational, or other?)	•Recruiting managers are aware of their trust’s recruitment and selection policies which emphasise equality for candidates	“we’re wanting diversity, you want people to represent the communities in which they serve.” [Int 2: NRM band 7 White M]
	• Recruiting managers suggested that workforce diversity is desirable but not essential	"[the local] population is BME [black and minority ethnic] and its very diverse […] It mirrors their background, mirrors what they see normally, and you know, it's easy to serve the population that you mirror.” [Int 12: NRM band 8 Black F]
	• Although recruiting managers feel it is important for the workforce to reflect the diversity of populations served, they do not have specific goals or targets for workforce diversity for their unit or organisation	–
2) Are recruiting managers involved in, or aware of, any workforce engagement and monitoring of diversity, or actions to identify or address diversity issues?	• Some recruiting managers were cautious about talking about the ethnicity of candidates and how to broach the subject of ethnic diversity in recruitment, indeed they felt talking about ethnicity went against the principles of equality	“Oh God no, not at all….No, not at all. It doesn’t factor at all, to be honest with you, we’re all from somewhere else, do you know what I mean, I’m Irish… so no. " [Int 4: NRM band 8 White F]
	• Recruiting managers were unlikely to talk about race equality or to openly discuss ethnicity issues in relation to recruitment	“In my years of doing interviewing appointment that I could honestly say that I’ve [n]ever experienced that there is any discrimination at any point in the interview process.” [Int 5: PRM band 8 White F]
	• Some recruiting managers were fearful that they might be accused of being racist if attention was directed towards the ethnicity of the candidates they have appointed	"I've got to be so careful what I say, when I came here the majority of my staff were black and probably Filipino, that has changed completely and I feel very conscious of that and I actually feel a little fearful and I'm surprised no one has pulled the racist card and I have a very black but also older workforce, but they don’t want to further themselves […] I've recruited 50 new members of staff in the last 12 months and lots of them have been young, white dynamic nurses.” [Int 11: NRM band 8 White F]
3) Are recruiting managers aware of, or engaged in, any monitoring or assessment of ethnic diversity in recruitment?	• Recruiting managers were unaware of ethnic inequalities in job appointments in their trust and when they were made aware of the statistics, they wrongly attributed discrepancies to a perceived weakness in ethnically diverse candidates	“I think those candidates who find it difficult, in all honesty, would have to look at themselves to say is there, issues within themselves?” [Int 2: NRM band 7 White M]
		“In all honesty I don’t know whether it was a bad year they [referring to ethnically diverse candidates] qualified and there was so many people looking for jobs, maybe they left it too late, maybe they failed their drug test”. [Int4: NRM band8 White F]
		“It’s about the individual, I think if you prepare yourself, you come in, you give us what we want, there’s no way, because to turn somebody down you have to be 100%, you know, I can’t say ‘just because the way you look’ I’m not going to offer you a job’ [Int 9: NRM band 8 Asian M]
	• Recruiting managers were not responsible for monitoring or feeding back about ethnicity in recruitment and some wrongly conflated issues of racial inequalities with a perceived deficit in the training of overseas nurses	“I know, there are issues with some overseas training courses and whether they're [British graduates from ethnically diverse backgrounds] potentially getting lumped in with that and actually whether there's been a judgement made that actually they're not coming from an English [university].” [Int 8: PRM band 8 White M]
	• All recruiting managers were able to make informal assessments and judgements about the ethnic diversity of their unit staff and the candidates they have appointed, they were less likely to be able to make a judgement about the ethnicity of applicants to posts or to identify underrepresentation in applicants to jobs	–
4) How far do recruiting managers value candidates as unique individuals that have different skills and abilities, assets, potential and strengths, to serve diverse patient groups and populations?	• Recruiting managers said that they valued candidates as individuals but there was no time or resources to offer support to new recruits	“It’s just not a luxury we have […] we need somebody to come in and just get on with the job, not have lots and lots of supervision, training, and things.” [Int 8: NRM band 8 White M]
	• For all recruiting managers, a policy of equality in recruitment meant adhering to job selection criteria for shortlisting and using and using candidate scoring systems based on technical competencies	
	• Recruiting managers were often responsible recruiting large numbers of staff in a short period of time alongside having clinical management duties, which could result in pressure to appoint	“Some of them stand out, you know, they’ve done additional pieces of work, or they’ve travelled, or they’ve done, you know, and they’ve got keen interest in different things” [Int 12: NRM band 8 Black F]
	• Selection processes including technical exercises, group interviews, visits to the unit and individual interviews were used to assess candidates and make judgements about suitability for the job	"… have they been to Cambodia voluntarily, you know, working in an orphanage or something like that? Have they been to Africa? Or have they helped out in the London Marathon? Have they done something additional outside, maybe voluntarily?” [Int 3: PRM band 8 White M]
	• Because of a pressurised competitive system, recruiting managers said they felt they made judgements about which candidates demonstrated experiences or skills above and beyond specified job criteria	“appearance is another thing you take into consideration, if somebody comes in scruffy, low top, short dress, we do mark them down” [Int1: NRM band 7 Black F] “they just walk in with a t-shirt on, just you know, it looks a bit scruffy” [Int6: NRM band 7 Black F]
	• Recruiting managers said they judged candidates on their presentation at interview including their appearance and professionalism, some suggested that candidates from ethnic minority backgrounds should be made aware that they were being judged by their appearance as a professional	“I think it would help in, you know, to give that sort of guidance to them, you know, professional trends, you know, how they face so that students are aware when this is what you will be expected, some sort of preparation.” [Int 7: NRM band 8 Asian F]
5) How are the interests or concerns of underrepresented ethnically diverse groups heard, voiced, or advocated for in relation to recruitment practices?	• Some recruiting managers were sceptical about equity in recruitment as they perceived it as undermining equality, or showing favouritism or positive discrimination	“I don’t agree with positive discrimination, I think I treat everyone equally.” [Int 11: NRM band 8 White F]
	• Some recruiting managers said they openly try to encourage ethnically diverse candidates to apply to posts, by including statements about diversity in job advertisements	"I’m thinking, okay, that’s obviously not an English name, although I’m sort of judging them on the name but it might be they’re married and I try thinking, okay, is there anything on this paper that can actually get me to get this person to come and be shortlisted” [Int 1: NRM band 7 Black F]
	• Some recruiting managers said they informally encourage ethnically diverse candidates to apply by informing individuals known to them about upcoming posts and how to apply	“I would try my utmost first to get that person and obviously if they don’t come up to scratch then I won’t shortlist them… I'd will them to do well, I'm always willing them to do well." [Int1: NRM band 7 Black F] [candidates from a named university] “they’re basically guaranteed an interview.” [Int 2: NRM band 7 White M]
	• None of the recruiting managers reported equity interventions or initiatives designed to encourage underrepresented ethnically diverse groups to apply, or to address barriers to recruitment or selection, indeed candidates were more likely to be offered an interview based on the university they were attending rather than their grade or experience	–
6. How far do recruiting managers see a need for change, better understanding, relationships, or resilience building between groups or organisations to support fairer recruitment and retention of human potential?	• Although some recruiting managers sense ethnically diverse candidates may have to work harder to be the strongest candidate on the day, not everyone perceives ethnic inequality in first job success to be wrong or problematic for their trust	“I honestly don’t know, it’s not something I’ve ever thought about [ethnic inequalities in job success] or come across.” [Int 8: NRM band 8 White M]
	• Some recruiting managers are perplexed and want more information and statistics to better understand ethnic inequalities in first job success	“Anecdotally yes, yes, it doesn't surprise me, I can't tell you why … there were girls from BME groups, and they were pretty good … we had exceptional talent … in terms of the, of saying why others don't get the job, I have no idea, no, no because the ones I've seen were really good.” [Int 12: NRM band 8 Black F] “It’d be interesting to know what the actual ethnic minority and whether that has an impact on whether they’re then selected or not as I say, because that’s not something that we’re aware of” [Int10: NRM band 8 White F]
	• One recruiting manager (from an ethnically diverse background) suggested that candidates needed to realise they would need to show they were better prepared and better able for the post than their white counterparts	“Obviously having come up from that pool of candidates myself I’ve always been aware that I will have to present myself as more able, I don’t know how to sort of put it correctly, maybe there is no correct way, I know potentially I will have to be a stronger candidate […] I think that still stands in some regard. [Int 6: NRM band 7 Black F]
	• One recruiting manager (from an ethnically diverse background) spoke about racial bias in recruitment	"We’re supposed to be, or we are a progressively more integrated society, there’s no two ways about that one, but some things intrinsically are there, way back sometimes beyond what people actually are consciously thinking, and sometimes that affects their decision making” [Int 6: NRM Black F]

Our critically informed analysis identified three issues relating to this question. First, all recruiting managers are aware of their trust’s recruitment and selection policies, which, in accordance with national policies and guidance, emphasise equality for candidates. According to recruitment policies equality was enacted in practices such as “everyone has the same chance and it’s the same criteria for everybody” [Int4: NRM band 8 White F], “the application form (is) blinded to age and their name” [Int6: NRM band 7 Black F], and “shortlisting criteria” [Int3: PRM band 8 White M] used as part of “a pointing and scoring system” [Int5: PRM band 8 White F] with “the people that have scored the highest” being successful [Int10: NRM band 8 White F]. Second, recruiting managers suggested that workforce diversity is desirable but not essential. This was evidenced in comments about the staff diversity should reflect the diversity of patient populations. They expressed this staff diversity as “reflecting” [Int2: NRM band 7 White M] or “mirroring” patient populations [Int12: NRM band 8 Black F]. Third, although recruiting managers feel it is important for the workforce to reflect the diversity of “our multicultural populations” [Int2: NRM band 7 White M], they do not have specific goals or targets for workforce diversity for their unit or organisation. Participants were unlikely to elaborate on why they believe diversity is a positive feature of the workforce, or to make a link between staff diversity and the delivery of high-quality care to diverse patient groups.

2 Are recruiting managers involved in, or aware of, any workforce engagement and monitoring of diversity, or actions to identify or address diversity issues?

Three issues were identified in relation to this question. First, some recruiting managers were cautious about talking about ethnicity or unsure how to broach the subject of ethnic diversity in recruitment without seeming racist [Int11: NRM band 8 White F] or being unfair [Int5: PRM band 8 White F]. Second, recruiting managers were unlikely to talk about race equality (none used this term) or to openly discuss ethnicity issues in relation to recruitment. Indeed, some recruiting managers conveyed complete confidence in their adherence to organisational policies for recruitment and selection and therefore they were “in no way discriminatory” because they “treat everyone exactly the same” [Int5: PRM band 8 White F]. Third, some recruiting managers were “very conscious” and “afraid” [Int11: NRM band 8 White F] that they might be accused of being racist if attention was directed towards the ethnicity of the candidates they have appointed. As one account from a recruiting manager in nursing showed how ambiguity about diversity had allowed her to gradually replace an older ethnically diverse workforce, who she feels are less ambitious. She states, *“*I've recruited 50 new members of staff in the last 12 months and lots of them have been young, white dynamic nurses.” [Int 11: NRM band 8 White F] (see Table 2 for quote in context).

3 Are recruiting managers aware of, or engaged in, any monitoring or assessment of ethnic diversity in recruitment?

Issues in relation to this question were firstly, recruiting managers were unaware of ethnic inequalities in job appointments in their trust and when they were made aware of the statistics, they attributed discrepancies to a perceived weakness in ethnically diverse candidates (cultural deficit model [35]). Lack of available information about ethnicity and workforce diversity led some recruiting managers to make prejudiced stereotypes of ethnically diverse candidates as being less able [Int2: NRM band 7 White M], less “confident or prepared [Int9: NRM band 8 Asian M], unprofessional, or not career focused [Int11: NRM band 8 White F] or external factors (such as incomplete application, late applications, or failed technical assessments) [Int4: NRM band 8 White F] that might account for the varying recruitment statistics for diverse candidates*.* Second, recruiting managers were not responsible for monitoring or feeding back about ethnicity in recruitment and some conflated issues of racial inequalities with a perceived deficit in the training of overseas nurses. One recruiting manager suggested that there could be prejudices against British ethnically diverse candidates because “a judgement is being made that they’re not coming from an English” university [Int8: PRM band 8 White M] and there might be an assumption that overseas qualifications are of a lesser quality than UK qualifications. This view demonstrates a lack of valuing of the unique assets that migrants contribute to the workforce, as has been noted in previous research in the NHS [9]. Third, all recruiting managers were able to make informal assessments and judgements about the (unrepresentative) ethnic diversity of their unit staff and the candidates they have appointed, however, they were less likely to make a judgement about the ethnicity of applicants to posts or to identify underrepresentation in applicants to jobs.

4 How far do recruiting managers value candidates as unique individuals that have different skills and abilities, assets, potential and strengths, to serve diverse patient groups and populations?

This question identified six issues. First, recruiting managers said that they valued candidates as individuals, but there was no time or resources to offer specific support to new recruits in terms of supervision or training beyond standard induction programmes [Int8: PRM band 8 White M]. This meant they were more likely to appoint “somebody to come in and just get on with the job” [Int8: PRM band 8 White M]. Second, for all recruiting managers, a policy of equality in recruitment meant adhering to job selection criteria for shortlisting and using candidate scoring systems based on technical competencies. Third, recruiting managers were often responsible for recruiting large numbers of staff in a short period of time alongside having clinical management duties, which could result in pressure to appoint. Fourth, selection processes including technical exercises, group interviews, visits to the unit and individual interviews were used to assess candidates and make judgements about suitability for the job. Fifth, recruiting managers said that having to decide between suitably qualified candidates meant that they might need to look for any other outside interests [Int12: NRM band 8 Black F] travel or voluntary work experiences [Int3: PRM band 8 White M]. There was no suggestion that candidates from ethnically diverse backgrounds might have other unexplored, but equally useful, life experiences that would benefit the workforce, or that they might be disadvantaged from engaging in extra curricula activities that might incur time and expense [36]. Overall, there was a lack of transparency about these additional ‘performance’ criteria by which candidates were being judged. Sixth, recruiting managers said they judged candidates on their personal presentation at interview including their appearance. Some recruiting managers tended to equate appearance with professionalism [see Table 1, examples from Int:1,6,7] and were influenced by their opinion of clothing choices, despite the fact that all staff would be expected to wear a uniform if appointed.

5 How are the interests or concerns of underrepresented ethnically diverse groups heard, voiced, or advocated for in relation to recruitment practices?

There were four issues that related to this question. First, some recruiting managers were sceptical about equity in recruitment as they perceived it as undermining equality, or showing favouritism, or conflated equity with positive discrimination [Int11: NRM band 8 White F]. Second, some recruiting managers said they generally “try to encourage” [Int1: NRM band 7 Black F] ethnically diverse candidates to apply to posts by including statements about diversity in job advertisements. Third, some recruiting managers said they personally encourage specific ethnically diverse candidates to apply by “informing individuals” [Int1: NRM band 7 Black F] known to them about upcoming posts and how to apply. Fourth, none of the recruiting managers reported equity interventions or initiatives designed to encourage underrepresented ethnically diverse groups to apply, or to address barriers to recruitment or selection, indeed candidates were more likely to be offered or “guaranteed an interview” [Int2: NRM band 7 White M] based on the university they were attending rather than their degree/grade or experience. While recruiting managers tended to see these practices as ensuring there is a competitive pool of candidates, these biases could be conceptualised as ‘opportunity hoarding’ [34] (not being transparent about opportunities that are available).

6) How far do recruiting managers see a need for change, better understanding, relationships, or resilience building between groups or organisations to support fairer recruitment and retention of human potential?

Four issues were evident in relation to this question. First, although some recruiting managers sense that ethnically diverse applicants need “to be a stronger candidate” [Int6: NRM band 7 Black F] than their white counterparts, not everyone perceives ethnic inequality in first job success to be problematic for their trust. Second, some recruiting managers said they were unaware [Int8, Int10, Int12] or “had no idea why” and “it would be interesting to know” the reasons behind ethnic inequalities in first job success [Int10: NRM band 8 White F]. Reasons offered for racial discrepancies included competition for places because of the prestige of an organisation or clinical field but these assumptions overlook structural discrimination. Third, one recruiting manager in nursing (from an ethnically diverse background) [Int6: NRM band 7 Black F] suggested that candidates needed to realise they would need to show they were better prepared and “more able” for the post and “a stronger candidate” than their white counterparts. Fourth, only this same recruiting manager spoke about personal experience of racial bias in recruitment. This recruiting manager [Int6: NRM band 7 Black F] suggested, older generations from migrant communities implicitly knew they would have to work harder to demonstrate their abilities and strengths, but second-generation migrant candidates are not as aware “some things are intrinsically there” and “that affects their decision-making”.

In summary, while many of the recruiting managers acknowledged that racial discrimination might occur, they are complicit within a system that does not question unjust recruitment practices. Instead, they erroneously attributed racial discrepancies to a few imagined racist colleagues with recruitment responsibilities, or the misperceived weaknesses or failings of ethnically diverse candidates. None questioned recruitment practices or the accumulation of barriers to appointment for ethnically diverse candidates. There was no suggestion from recruiting managers that it was within the remit, or responsibility of their role, or their employing organisation to support ethnically diverse candidates to attain jobs they are trained and qualified for. The discussion looks at these findings in the broader context of what is already known.

## Discussion

This critically informed study helps to explain the reasons behind underrepresentation of ethnically diverse candidates in first NHS healthcare jobs post qualification [22] and why white applicants are 1.61 times more likely to be appointed from shortlisting than ethnically diverse applicants [12]. A lack of guidance on diversity and equity is creating uncertainty for recruiting managers about whether diversity and equity should be specific goals or targets for recruitment. It is unclear and how these concepts relate to existing recruitment and selection policies that emphasise equality. The recruiting manager is in the director’s chair having to make decisions about appointments that comply with the organisation’s expectations and at the same time dealing with anxiety about their personal performance and not wanting to appear to be racist by recognising candidate ethnicity as being an issue. This caution about talking about ethnicity issues within the NHS, is limiting progress on race equality and upholding the cultural deficit model [35] (the mistaken assumption that candidates are lacking in some way). Recruitment approaches need to be better informed about ethnic inequalities in job appointments and support provided for recruiting managers. Such understandings can inform organisational strategies to provide a “framework” [28] for equity, such as engaging with the workforce to discuss ethnicity issues, monitoring, and systems for feeding back about ethnicity issues.

All recruiting managers were able to make informal assessments and judgements about the ethnic diversity of their unit staff and the candidates they have appointed. They were less likely to be able to make a judgement about the ethnicity of applicants to posts or to identify underrepresentation in applicants to jobs. There are clearly points of confusion and unhelpful stereotyping that need to be addressed, such as the conflation of racial inequalities in first job success as an issue relating to a perceived deficit in the training of overseas nurses.

Our critical stance on this ‘equality theatre’ draws back the curtain on the façade of equality in recruitment and the “ironical effects” [28] which discriminate against ethnically diverse candidates in multiple ways. Our approach emphasises organisational learning and development [29] and argues for approaches to address organisational deficits that will promote equalities in first job success, rather than laying blame at the door of individuals such as recruiting managers or candidates. It is important to note that the two NHS trusts in this study were performing better than average on WRES indicators (1.3 and 1.4 compared to 1.6 nationally) on race equality in appointments from shortlisting [12]. As our wider programme of research has shown, poor performance on racial equality is not simply a matter of being refused entry on to the stage, some actors will not even go to certain theatres for auditions because they sense they will not ‘get in’ or ‘fit in’ to particular organisations or units [23].

The study has identified some of the barriers to employment success for ethnically diverse candidates. These include the time pressures on managers associated with selecting and appointing staff from large numbers of applicants. Strategies which aim to make recruitment more efficient (technical exercises, group interviews and structured interviews) may inadvertently create a narrow set of criteria that might further advantage whiteness and associated values. It can take extra time and understanding to appreciate the diverse skills and experiences that a wider pool of candidates might contribute above and beyond job criteria. Recruitment strategies need to be developed that help to recognise diverse skills and experiences, such as contextualised recruitment, for an example see Hammond et al [19].

Physical appearance of candidates is a barrier to employment that cannot be ignored. Recruiting managers said they judged candidates on their presentation at interview including their personal and professional appearance. The findings from this study indicate what was ‘professional’ was rather rigidly interpreted. Employers and recruitment processes need to critically examine definitions of professional appearance and think what might matter more to patients and patient outcomes once a person is in uniform.

Owing to the fact that this study is located in a UK policy context, and the data are derived from two London trusts in 2015, we have been cautious to offer solutions for health provider organisations more widely. Although changes may or may not have occurred since 2015 comparable WRES data suggest a similar situation. That said, we have built a robust argument for NHS trusts to integrate equity and diversity into recruitment and selection policies for the benefit of patient outcomes [13], service efficiency [1] and workforce sustainability [2–6, 8, 9]. The issue of leadership was not a question in the interviews with recruiting managers. Nevertheless, leadership is required to critically review healthcare recruitment practices across all levels to drive change and ensure that recruiting managers feel equipped to try new ways of working.

Our findings suggest that recruiting managers should be better informed about equity in recruitment (description in Box 1) and how this differs from positive discrimination. Indeed, training for recruitment managers should now focus on equity and diversity rather than equality, including information about equity interventions and initiatives to encourage underrepresented ethnically diverse groups to apply, or to address barriers to recruitment or selection. This should be backed up by more information and statistics for recruiting managers to better understand ethnic inequalities in first job success.

There are clearly significant challenges and changes needed to enable recruitment processes to attain the value of an ethnically diverse workforce as a source of resilience, enrichment, and creativity [6, 20]. Although the literature argues for new types of contextualised recruitment [19], allyship, cross-cultural mentorship [30], and networking to support equity [34], there is a lack of practical guidance about how to implement such strategies in the context of the performance politics of the NHS and secondly how much time to devote to equity or how to monitor the effects on recruitment or workforce diversity. Transitions through education and into employment need to be understood in the wider context of societal ethnic inequalities [26]. This requires partnership working and a strategic overview which can be supported by the Healthcare Workforce Equity + Diversity Lens we have developed.

## Conclusion

Adherence to recruitment and selection policies, which aim to support equality through standardisation and anonymisation, is limiting workforce diversity and creating barriers for ethnically diverse candidates to attain jobs that they are trained and qualified for. The Healthcare Workforce Equity + Diversity Lens we have developed can help to ‘raise the curtain on the equality theatre’ by focusing on six dimensions of diversity and equity. Healthcare employers can seek to address the reasons behind racial inequalities and barriers to employment for ethnically diverse candidates and critically examine their recruitment policies to identify any areas that may inadvertently discriminate. Employers can work with a wide range of stakeholders at this important point of transition, including patient groups and universities, to understand how their organisation may create a more inclusive approach to recruitment to first healthcare job.

## Supplementary Information

**Additional file 1. **Key search terms (/Medical Subject Headings MeSH).

**Additional file 2. **Healthcare workforce equity + diversity bibliography.

## Data Availability

Survey data and anonymised verbatim transcripts are stored in accordance with the R&D policies of Kingston University.
