# Supplementary material for: “Raising the curtain on the equality theatre”: a study of recruitment to first healthcare job post-qualification in the UK National Health Service
Source: Hum Resour Health. 2022 Jul 8;20:57. doi: 10.1186/s12960-022-00754-9 (PMC9264517; doi:10.1186/s12960-022-00754-9)
Supplement: Supplementary file 1 — Additional file 1. Key search terms (/Medical Subject Headings MeSH). [file 12960_2022_754_MOESM1_ESM.docx]

Additional file 1

Appendix 1: Key search terms (/Medical Subject Headings MeSH)

| **Concept** | **Workforce Diversity concept** | **Social justice concept** | **Equity concept** |
| --- | --- | --- | --- |
| MeSH terms (2022) | **Health Care**  Health Care Facilities, Manpower, and Services  Health Care Economics and Organizations  Health Services Administration  Health Care Quality, Access, and Evaluation  **Disciplines and occupations**  Health occupations  **Named groups**  Students, Health Occupations | - | - |
| Key terms | **Diversity**  BME/BAME (Black, Asian, minority)  Diversity  Ethnic/ethnicity  Ethnically diverse  **Workforce**  Career  Candidate  Employee  Employment  Interview  Job  Post-qualification  Recruitment  Selection  Staff retention  Staff skills  Talent  Workforce  Workforce planning | Disparity/disparities  Equality gap  Equity gap  Equality of opportunity  Human potential  Inequality/inequalities  Life chance  Social justice  Social mobility  Racial justice  Race/race equality  Racialized | Affirmative action  Equality  Equity intervention  Induction  Mentorship  Positive action  Positive intervention  Preceptorship  Redistribution  Role model |
