# Supplementary material for: “Raising the curtain on the equality theatre”: a study of recruitment to first healthcare job post-qualification in the UK National Health Service
Source: Hum Resour Health. 2022 Jul 8;20:57. doi: 10.1186/s12960-022-00754-9 (PMC9264517; doi:10.1186/s12960-022-00754-9)
Supplement: Supplementary file 2 — Additional file 2. Healthcare workforce equity + diversity bibliography. [file 12960_2022_754_MOESM2_ESM.docx]

**Additional file 2**

**Appendix 2: Healthcare Workforce Equity + Diversity bibliography**

**Workforce diversity goals & policies**

A Voice for the Reduction of Poverty Nashville. (2021) Equity lens. https://avoicenashville.com/equity-lens

Alderwick H, Charles A, Jones B and Warburton W (2017) Making the Case for Quality Improvement: Lessons for NHS boards and leaders. The King’s Fund. www.kingsfund.org.uk/publications/making-case-quality improvement.

Anderson M, O'Neill C, Macleod Clark J, Street A, Woods M, Johnston-Webber C, Charlesworth A, Whyte M, Foster M, Majeed A, Pitchforth E, Mossialos E, Asaria M, McGuire A. (2021) Securing a sustainable and fit-for-purpose UK health and care workforce. Lancet. 22;397(10288):1992-2011. doi: 10.1016/S0140-6736(21)00231-2. Epub 2021 May 6. PMID: 33965066.

Chartered Institute of Personnel and Development (2015) Quotas and Targets: How do they affect diversity progress? Chartered Institute of Personnel and Development.

Commission on Race and Ethnic Disparities (2021) Commission on Race and Ethnic Disparaties: The report. Commission on Race and Ethnic Disparities.

Department of Health and Social Care (2021b) NHS Constitution for England. Department of Health and Social Care.

Department of Health and Social Security (1980) Inequalities in Health: Report of a research working group. HMSO.

Equality and Human Rights Group, Department of Health (2006) Equality and Human Rights in the NHS: A guide for NHS boards. Department of Health.

LaVeist TA, Pierre G. (2014) Integrating the 3Ds--social determinants, health disparities, and health-care workforce diversity. Public Health Rep. Jan-Feb;129 Suppl 2(Suppl 2):9-14. doi: 10.1177/00333549141291S204. PMID: 24385659; PMCID: PMC3863706.

NHS England and NHS Improvement (2021) ‘Our NHS people profession: big conversation’. OurNHSPeopleProfession.org.

NHS Standard Contract Team, NHS England (2021) Full-length NHS Standard Contract 2021/22 (Particulars, Service Conditions, General Conditions). NHS England.

Philanthropic Initiative for Racial Equity (2021) Racial Justice Lens. https://racialequity.org/

Race Matters Institute (2021) Racial Equity Organizational Assessment. https://viablefuturescenter.org/racemattersinstitute/

Siva N (2009) ‘Race relations in the UK’s National Health Service’, The Lancet 373(9679), 1935–6.

Stanford FC. (2020) The Importance of Diversity and Inclusion in the Healthcare Workforce. J Natl Med Assoc. Jun;112(3):247-249. doi: 10.1016/j.jnma.2020.03.014. Epub 2020 Apr 23. PMID: 32336480; PMCID: PMC7387183.

The Nonprofit Association of Oregon (2018) Equity & Inclusion Lens Guide. https://nonprofitoregon.org/sites/default/files/NAO-Equity-Lens-Guide-2019.pdf

Wilbur K, Snyder C, Essary A, Reddy S, Will, K, Saxon, M. (2020) Developing Workforce Diversity in the Health Professions: A Social Justice Perspective, Health Professions Education, 6, 2, 222-229. ISSN 2452-3011, https://doi.org/10.1016/j.hpe.2020.01.002.

WRES Implementation Team (2019) A Model Employer: Increasing Black and minority ethnic representation at senior levels across the NHS. NHS England.

WRES Implementation Team (2020) NHS Workforce Race Equality Standard: 2019 data analysis report for NHS trusts. NHS England.

WRES Implementation Team (2021a) NHS Medical Workforce Race Equality Standard (MWRES): 2020 data analysis report for the NHS medical workforce. NHS England.

WRES Implementation Team (2021b) Workforce Race Equality Standard: 2020 data analysis report for NHS trusts and clinical commissioning groups. NHS England.

**Workforce engagement & monitoring**

Be Loved Community Equity Lens Map. (2021) Equity Audit and Equity Lens. https://www.wearebeloved.org/equity-audit-and-equity-lens-map-key-terms

Chartered Institute of Personnel and Development (2019) Diversity Management That Works: An evidence-based view. Chartered Institute of Personnel and Development.

Cohen J, Gabriel B, Terrell C. (2002) The case for diversity in the health care workforce. Health Aff (Millwood). Sep-Oct;21(5):90-102. doi: 10.1377/hlthaff.21.5.90. PMID: 12224912.

Dawson J (2009) Does the Experience of Staff Working in the NHS Link to the Patient Experience of Care? An analysis of links between the 2007 acute trust inpatient and NHS staff surveys. Institute for Health Services Effectiveness, Aston Business School.

Dawson J (2018) Links between NHS Staff Experience and Patient Satisfaction: Analysis of surveys from 2014 and 2015. NHS England.

Department for Business, Energy and Industrial Strategy (2017) Race in the Workplace: The McGregor-Smith review. Department for Business, Energy and Industrial Strategy.

Naqvi H (2020) ‘It’s no longer enough to know, we must act: workforce race inequality in the NHS’. www.kingsfund.org.uk/blog/2020/07/workforce-race-inequality-nhs.

NHS Digital (2021a) ‘Hospital and Community Health Services (HCHS) workforce statistics: ethnicity by staff group and grade, in NHS trusts and CCGs in England, September 2009 to December 2020, headcount’. Available in “Equality and diversity in NHS Trusts and CCGs December 2020” dataset. https://digital.nhs.uk/data-and-information/publications/statistical/nhs-workforce-statistics/december-2020.

NHS Digital (2021b) ‘Hospital and Community Health Services (HCHS) workforce statistics: gender by staff group and grade, in NHS trusts and CCGs in England, September 2009 to December 2020, headcount’. Available in “Equality and diversity in NHS Trusts and CCGs December 2020” dataset. https://digital.nhs.uk/data-and-information/publications/statistical/nhs-workforce-statistics/december-2020.

NHS England. (2015) The NHS Workforce Equality Standard – the defined metrics, Leeds.

NHS Survey Coordination Centre (2021a) NHS Staff Survey 2020: National dashboards. NHS Survey Coordination Centre.

NHS Survey Coordination Centre (2021b) NHS Staff Survey: National WDES and WRES metrics data 2020. NHS Survey Coordination Centre.

Jackson CS, Gracia JN. (2014) Addressing health and health-care disparities: the role of a diverse workforce and the social determinants of health. Public Health Rep.129 Suppl 2(Suppl 2):57-61. doi:10.1177/00333549141291S211.

Rolewicz and Spencer (2020) "Chart of the week: Black NHS staff are underrepresented in senior management roles". Nuffield Trust. www.nuffieldtrust.org.uk/resource/chart-of-the-week-black-nhs-staff-are-underrepresented-in-senior-management-roles.

Ross S, Jabbal J, Chauhan K, Maguire D, Randhawa M and Dahir S (2020) Workforce Race Inequalities and Inclusion in NHS Providers. The King’s Fund. www.kingsfund.org.uk/publications/workforce-race-inequalities-inclusion-nhs.

The Salvation Army Racial Equity Lens. (2004) https://s3.amazonaws.com/usc-cache.salvationarmy.org/f45b2004-9e73-44a1-9102-159ee6387b1d_RACIAL+EQUITY+LENS.jpg

**Recruitment monitoring**

DiTomaso N. (2015) ‘Racism and discrimination versus advantage and favoritism: Bias for versus bias against’, Research in Organizational Behavior, 35(2015), pp. 57–77. doi: 10.1016/j.riob.2015.10.001.

Fanshawe S (2018) Diversity: The new prescription for the NHS. Good Governance Institute.

Government Alliance on Race and Equity Racial Equity Toolkit. (2016) Racial Equity Toolkit. https://www.michigan.gov/mdcr/-/media/Project/Websites/mdcr/racial-equity/racial-equity-toolkit.pdf?rev=d1b76b2b90b54328bd32dc4a141b9d76&hash=AAB0062A4E0284CD876C29C90934D8D9

Harris R, Ooms A, Grant R, Marshall-Lucette S, Chu CS, Sayer J, Burke L. (2013) Equality of employment opportunities for nurses at the point of qualification: an exploratory study. Int J Nurs Stud. Mar;50(3):303-13. doi: 10.1016/j.ijnurstu.2012.10.008. Epub 2012 Nov 8. PMID: 23141424.

Hammond J, Marshall-Lucette S, Davies N, Ross F, Harris R. (2017) Spotlight on equality of employment opportunities: A qualitative study of job seeking experiences of graduating nurses and physiotherapists from black and minority ethnic backgrounds. Int J Nurs Stud. Sep;74:172-180. doi: 10.1016/j.ijnurstu.2017.07.019. Epub 2017 Aug 1. PMID: 28800502.

Housing Development Consortium Race Equity Toolkit. (2014) https://www.housingconsortium.org/member-programs/racial-equity-inclusion/#Toolkit

Hunt V, Prince S, Dixon-Fyle S and Yee L (2018) Delivering through Diversity. McKinsey & Company.

Kline R (2015) Beyond the Snowy White Peaks of the NHS? Race Equality Foundation.

Kline R (2021b) ‘To boost diversity NHS leaders need to first understand their own biases’, Health Service Journal, 16 September.

Marshall-Lucette S, Chu C. (2013) Landing that first job. Nursing Standard, 27 (33): 72 17th April 2013.

NHS Employers (2021a) Inclusive Recruitment: Leading positive change. NHS Employers.

NHS Employers (2021b) Inclusive Recruitment: Supporting economic recovery. NHS Employers.

NHS Employers (2021c) Inclusive Recruitment: Increasing supply, widening access to employment and addressing inequality. NHS Employers.

Scobie S, Spencer J and Raleigh V (2021) Ethnicity Coding in English Health Service Datasets. Nuffield Trust. www.nuffieldtrust.org.uk/research/ethnicity-coding-in-english-health-service-datasets.

The Aspen Institute Education & Society Program Racial Equity Lens. (2018) Pursuing Social and Emotional Development Through a Racial Equity Lens: A Call to Action - The Aspen Institute https://www.aspeninstitute.org/publications/pursuing-social-and-emotional-development-through-a-racial-equity-lens-a-call-to-action/

Workforce Race Equality Standard Report (2020) https://www.england.nhs.uk/wp-content/uploads/2021/02/Workforce-Race-Equality-Standard-2020-report.pdf

**Contextualised recruitment**

Darling C and the WRES Implementation Team (2017) Workforce Race Equality: Case studies of good practice from non-NHS employers. NHS England.

Hammond, J.A., Lewis, K., White, H. and Bowman, D. (2012) Adjusting the Academy: Developing an Adjusted Entrance Criteria Scheme in a specialist Healthcare and Bioscience Higher Education Institution. Journal of Widening Participation and Lifelong Learning 13(3), pp 45-59

Hemmings N, Buckingham H, Oung C, Palmer W (2021) Attracting, supporting and retaining a diverse NHS workforce Research report, Nuffield Trust. London.

Kline R (2021a) No more tick boxes: a review on the evidence on how to make recruitment and career progression fairer. NHS East of England.

Läng J (2020) Analysis and Recommendations on Diversity of the Mental Health Workforce. National Workforce Skills and Development Unit.

Makoff-Clark A (2019) 'Is blind recruitment the secret to the perfect hire?' People Management.

NHS Employers (2015) Diversity and Inclusion: The power of research in driving change. NHS Employers, Imperial College London, enei (Employer’s Network for Equality and Inclusion) and North West Coast Academic Health Science Network.

NHS Providers (2020) ‘Podcast: Continuing the conversation: diversity in the NHS’. https://nhsproviders.org/resource-library/provider-bites/podcast-continuing-the-conversation-diversity-in-the-nhs.

Owoyemi O, Aakhus E. (2021) Underrepresentation in Oncology: Identifying and Addressing Structural Barriers. Oncologist. Aug;26(8):630-634. doi: 10.1002/onco.13771. Epub 2021 Apr 19. PMID: 33797155; PMCID: PMC8342590.

Seeleman C, Essink-Bot ML, Stronks K, Ingleby D. (2015) How should health service organizations respond to diversity? A content analysis of six approaches. BMC Health Serv Res. Nov 16;15:510. doi: 10.1186/s12913-015-1159-7. PMID: 26573437; PMCID: PMC4647506.

Smith TY. (2021) The time is now: A model for diversity recruitment and retention in emergency medicine training programs. AEM Educ Train. 29;5(Suppl 1):S126-S129. doi: 10.1002/aet2.10659. PMID: 34616986; PMCID: PMC8480503.

Qureshi I, Ali N, Randhawa G. (2020) British South Asian male nurses' views on the barriers and enablers to entering and progressing in nursing careers. J Nurs Manag.28(4):892-902. doi: 10.1111/jonm.13017. PMID: 32250005.

**Effective allyship**

Albany Medical College medical allyship module, reported in: Martinez S, Araj J, Reid S, et al. (2021) Allyship in Residency: An Introductory Module on Medical Allyship for Graduate Medical Trainees. Mededportal : the Journal of Teaching and Learning Resources. 17:11200. DOI: 10.15766/mep_2374-8265.11200. PMID: 34988287; PMCID: PMC8685188.

Belle RR, Cotton JL, Miller JS. Marginal mentoring: The effects of type of mentor, quality of relationship, and program design on work and career attitudes. Academy of Management Journal, 2000, 43(6), 1177–1194.

Bolden R, Adelaine A, Warren S, Gulati A, Conley H and Jarvis C (2019) Inclusion: The DNA of leadership and change. University of the West of England.

Care Quality Commission (2018) Equally Outstanding: Equality and human rights – good practice resource. Care Quality Commission.

Coghill Y (2020a) ‘Confronting race inequality with an open mind and open heart’, Evidence-Based Nursing blog, 22 November.

Coghill Y (2020b) ‘The seven A’s to becoming an authentic ally’. www.whatdotheyknow.com/request/703252/response/1692802/attach/16/185%2020%20The%20seven%20A%20s%20to%20becoming%20an%20authentic%20Ally.pdf?cookie_passthrough=1.

Dobbin F and Kalev A (2016) ‘Why diversity programs fail’, Harvard Business Review July to August. https://hbr.org/2016/07/why-diversity-programs fail.

Gill GK, McNally MJ, Berman V. Effective diversity, equity, and inclusion practices. Healthc Manage Forum. 2018 Sep;31(5):196-199. doi: 10.1177/0840470418773785. Epub 2018 Aug 16. PMID: 30114938.

Sealy R (2020) Action for Equality: The time is now. The Health and Care Women Leaders Network and NHS Confederation.

Surrey and Borders Partnership NHS Foundation Trust (2020) Workforce Disability Equality Scheme (WDES) Report and Action Plan. Surrey and Borders Partnership NHS Foundation Trust. www.sabp.nhs.uk/application/files/5316/0309/5754/WDES_Exec_Report_Sep_2020_Final.pdf.

UNISON (2021) ‘What could be done to improve representation, retention and progression opportunities for people of different ethnic backgrounds in public sector workforces? Evidence submission to the Commission on Race and Ethnic Disparities’.

University of Kent effective allyship resources. (2021) Allyship Resources - Equality, Diversity and Inclusivity - University of Kent https://www.kent.ac.uk/equality-diversity-inclusivity/effective-allyship

Women and Work All Party Parliamentary Group (2020) Inclusivity and Intersectionality: Toolkit and annual report 2019. GOV.UK.

**Transformative change**

Access, Equity, and Acceleration Unit equity lens. (2021) Equity Lens - Access, Equity, and Acceleration Unit (lausdaea.net) https://lausdaea.net/equity-lens/

Amin M, Till A and McKimm J (2018) ‘Inclusive and person-centred leadership: creating a culture that involves everyone’, British Journal of Hospital Medicine (London) 79, 402–7.

Beech J, Bottery S, Charlesworth C, Evans H, Gershlick B, Hemmings N, Imison C, Kahtan P, McKenna H, Murray R and Palmer B (2019) Closing the gap: key areas for action on the health and care workforce. The Health Foundation, The King's Fund and the Nuffield Trust.

Crenshaw K (1989) ‘Demarginalizing the intersection of race and sex: a Black feminist critique of antidiscrimination doctrine, feminist theory and antiracist politics’, University of Chicago Legal Forum 1989(1).

Delphin-Rittmon ME, Andres-Hyman R, Flanagan EH, Davidson L. (2013) Seven essential strategies for promoting and sustaining systemic cultural competence. Psychiatr Q. 84(1):53-64. doi: 10.1007/s11126-012-9226-2. PMID: 22581030.

Department of Health and Social Care (2021a) ‘Integration and innovation: working together to improve health and social care for all’. www.gov.uk/government/publications/working-together-to-improve-health-and-social-care-for-all/integration-and-innovation-working-together-to-improve-health-and-social-care-for-all-html-version.

Kaiser CR, Major B, Jurcevic I, Dover TL, Brady LM, Shapiro JR. (2013) Presumed fair: Ironic effects of organisational diversity structures. Journal of Personality and Social Psychology, 104(3), 504–519.

NHS Digital Strategic Data Collection Service (2021) ‘Workforce Race Equality Standard 2020: supporting data’. www.england.nhs.uk/publication/workforce-race-equality-standard-2020-supporting-data.

NHS Leadership Academy. (2021) ‘Inclusion, equality and diversity’. www.leadershipacademy.nhs.uk/resources/inclusion-equality-and-diversity.

Scott T, Mannion R, Davies HTO and Marshall MN (2003) ‘Implementing culture change in health care: theory and practice’, International Journal for Quality in Health Care 15, 111–18.

Sheffield Teaching Hospitals NHS Foundation Trust (2017) Improving Workforce Race Equality: A system wide approach. Sheffield Teaching Hospitals NHS Foundation Trust.

Snyder CR, Frogner BK, Skillman SM. (2018) Facilitating Racial and Ethnic Diversity in the Health Workforce. J Allied Health. 47(1):58-65. PMID: 29504021.

The Impact Foundry. (2021) Cultural Intelligence Unit - Impact Foundry - Training, Resources and Sustainability Consulting for Nonprofits https://impactfoundry.org/cultural-intelligence/
